# Supplementary material for: Plastic hepatocyte states limit liver cancer development
Source: Nat Commun. 2025 Nov 26;16:11647. doi: 10.1038/s41467-025-66568-0 (PMC12748961; doi:10.1038/s41467-025-66568-0)
Supplement: Supplementary file 1 — Supplementary Information [file 41467_2025_66568_MOESM1_ESM.pdf]

Antibody	Source	Identifier	Concentration Used
**Goat anti-mCherry**	Sicgen	AB0040-200	1:500
**Rabbit anti-RFP**	Rockland	600-401-379	1:100
**Rabbit anti-GFP**	Invitrogen	A11122	1:250 with TSA kit
**Rabbit anti-Sox9**	Millipore	AB5535	1:500
**Rabbit anti-Sox9**	Millipore	AB5535	1:2500 with TSA kit
**Goat anti-Osteopontin**	R&D	AF808	1:100
**Goat anti-Osteopontin**	R&D	AF808	1:100 with TSA kit
**Rabbit anti-CK19**	Abcam	Ab52625	1:100
**Rabbit anti-Hes1**	CST	11988	1:30 with TSA kit
**Goat anti-ECadherin**	R&D	AF648	1:500 with TSA kit
**Mouse anti-GS**	BD	610517	1:100
**Rabbit anti-HNF4**a	CST	3113	1:100
**Rabbit anti-Fah**	Abbomax	602-910	1:200
**Donkey anti-Rabbit 647**	Invitrogen	A31573	1:600
**Donkey anti-Rabbit Cy3**	Jackson Immuno	711-165-152	1:600
**Donkey anti-Rabbit 488**	Invitrogen	A21206	1:600
**Donkey anti-Rat Cy3**	Jackson Immuno	712-165-153	1:600
**Donkey anti-Goat 647**	Invitrogen	A21447	1:600
**Donkey anti-Goat 555**	Invitrogen	A21432	1:600
**Donkey anti-Goat 488**	Invitrogen	A21206	1:600
**Donkey anti-Mouse 405**	Jackson Immuno	715-475-151	1:200
**Donkey anti-Rabbit (HRP conjugated)**	Jackson Immuno	703-035-152	1:200 with TSA kit
**Donkey anti-Goat (HRP conjugated)**	Jackson Immuno	705-035-003	1:200 with TSA kit
**Rat anti-mouse CD326 (FITC)**	Biolegend	118207	0.25mg/million cells

Supplementary Table 1 Antibodies for Immunofluorescence

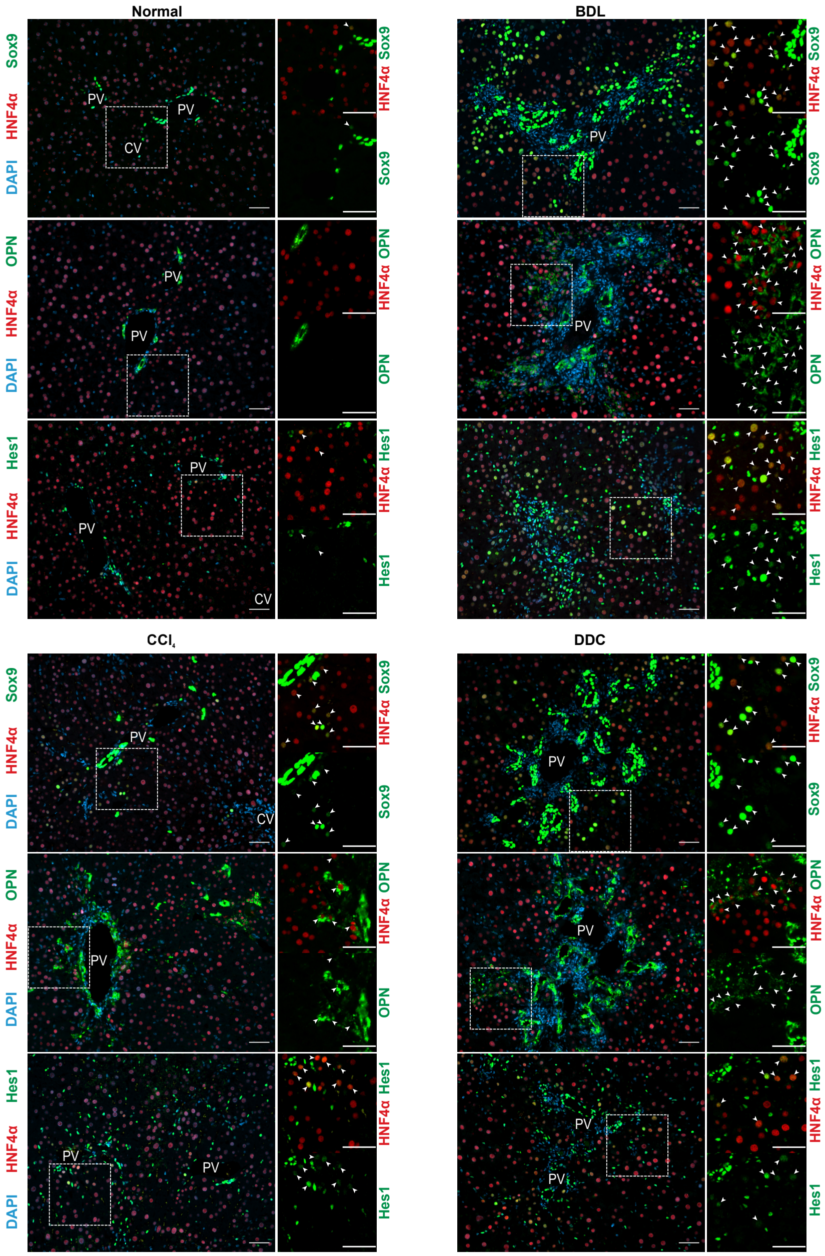

Supplementary Fig. 1. Plastic hepatocyte states are pervasive in various liver injury models.

Representative immunofluorescence images following co-staining of the hepatocyte marker HNF4a with the early BEC markers Sox9 and Osteopontin (OPN), or the direct Notch target gene Hes1 in the normal mouse liver or various mouse models liver damage. Arrowheads designate plastic hepatocytes positive for both HNF4a and the indicated BEC marker. PV, portal vein. CV, central vein. Scale bars, 50µm. BDL, bile duct ligation. CCl_4, chronic carbon tetrachloride. DDC, 0.1% 3,5-diethoxycarbonyl-1,4-dihydrocollidine diet.

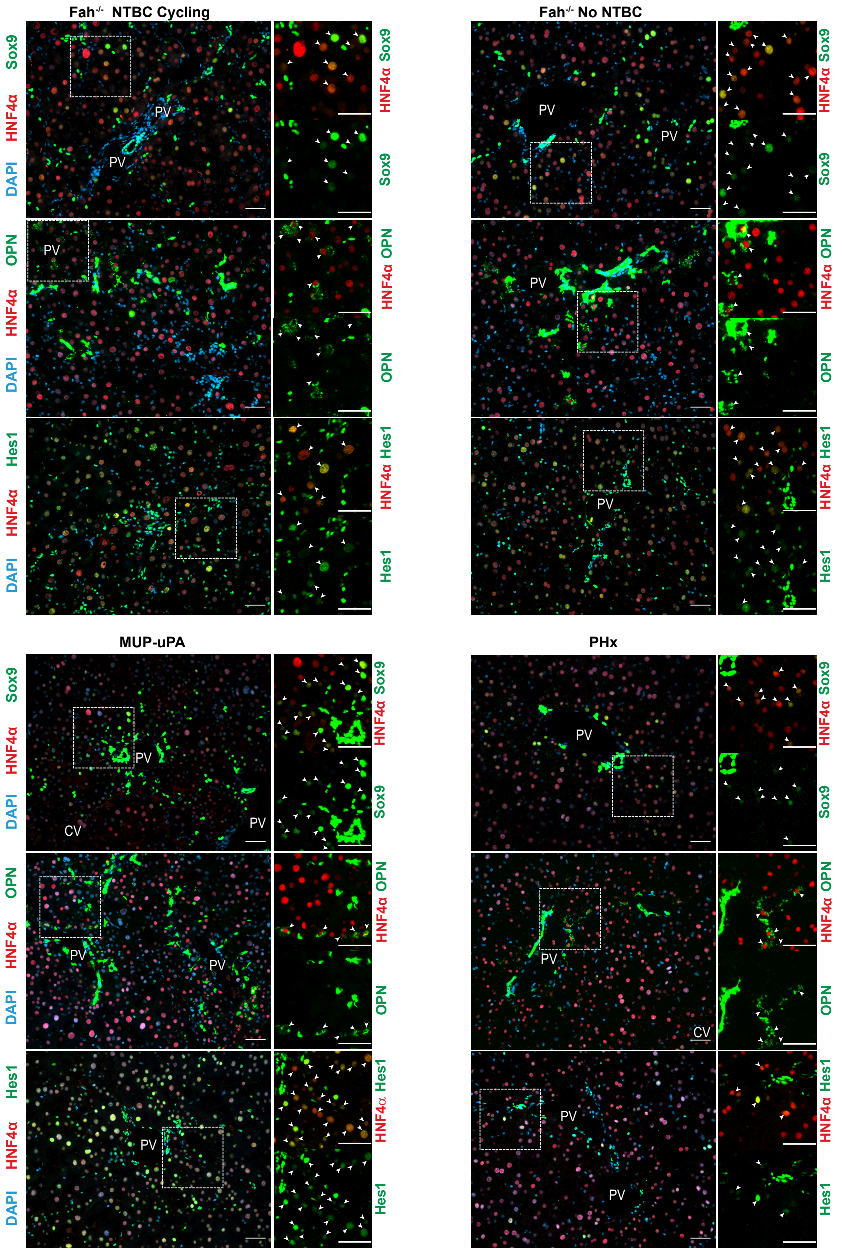

Supplementary Fig. 2. Plastic hepatocyte states are pervasive in various liver injury models. (continued)

Representative immunofluorescence images following co-staining of the hepatocyte marker HNF4a with the early BEC markers Sox9 and Osteopontin (OPN), or the direct Notch target gene Hes1 in the normal mouse liver or various mouse models liver damage. Arrowheads designate plastic hepatocytes positive for both HNF4a and the indicated BEC marker. PV, portal vein. CV, central vein. Scale bars, 50µm. $Fah^{-/-}$ NTBC cycling, Fumarylacetoacetate hydrolase$^{-/-}$ mice cycled off Nitisinone three times. $Fah^{-/-}$ no NTBC, Nitisinone withdrawn from $Fah^{-/-}$ mice continuously for 3 weeks. MUP-uPA, transgenic mice expressing urokinase-type plasminogen activator under control of the Major Urinary Protein promoter. PHx, 70% partial hepatectomy.

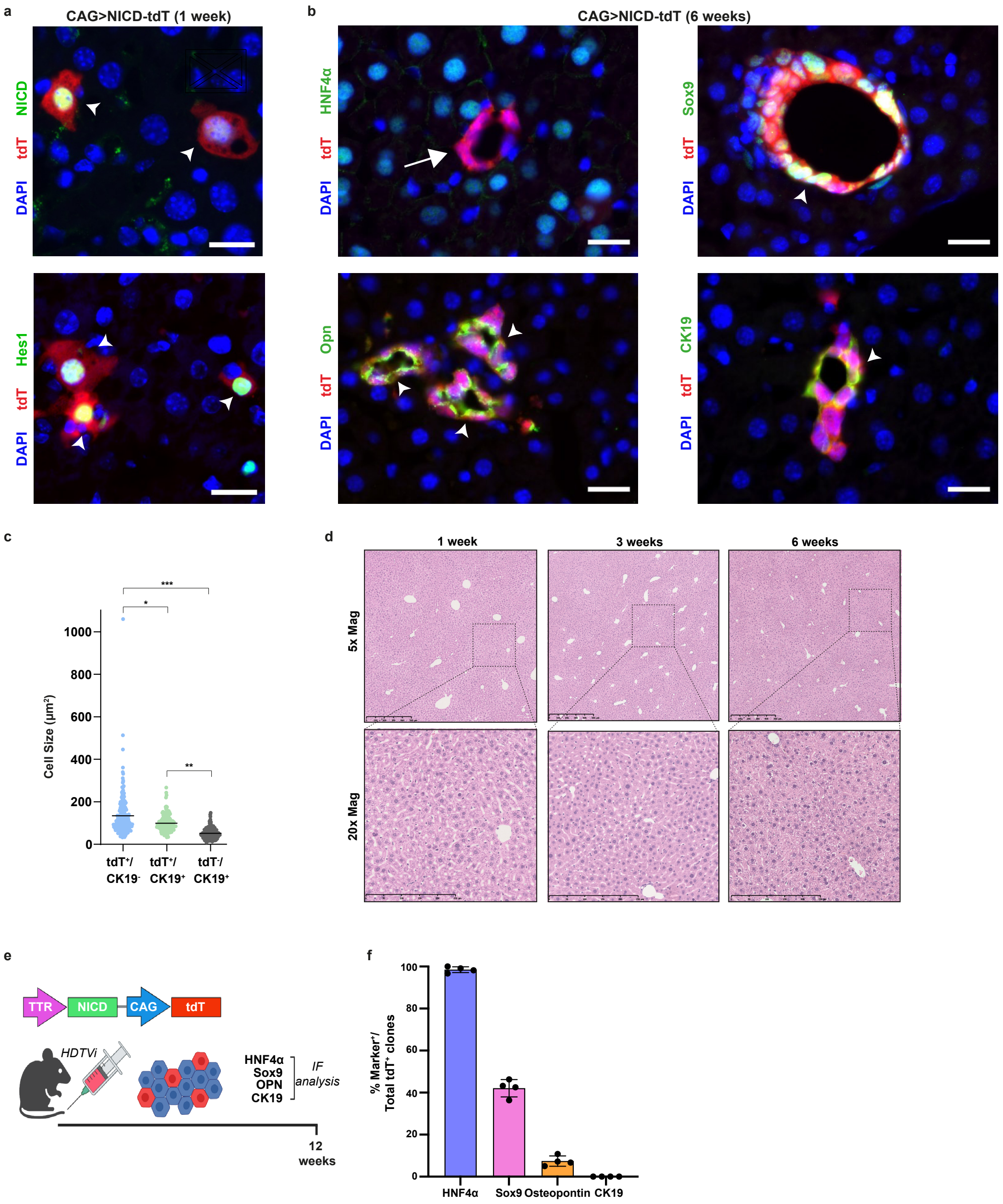

Supplementary Fig. 3. Further characterization of transposon-induced reprogramming.

a, Example immunofluorescence staining for Notch intracellular domain (NICD) or Hes1 and tdTomato (tdT) in liver sections from C57Bl/6 mice 1 week after HDTVi of CAG>NICD-tdT. Arrowheads indicate NICD^{+} tdT^{+} and Hes1^{+} tdT^{+} clones. n = 3 mice. **b,** Representative immunostaining for tdT, in combination with either HNF4a (hepatocyte marker), Sox9 and Opn (early BEC markers) or CK19 (mature BEC marker) on liver sections 6 weeks post-HDTVi with the CAG>NICD-tdT transposon, showing examples of formation of bile duct structures. Arrowheads denote tdT^{+} clones which express the indicated cell identity marker. Arrows indicate marker negative tdT^{+} clones. Scale bars, 20mm. n = 3 mice. **c,** Cell size analysis of tdT^{+} cells 6 weeks post-HDTVi of CAG>NICD-tdT samples, compared to normal tdT^{-}/CK19^{+} BECs. The mean of the data

for each cell type is denoted by solid black lines. Statistical significance was determined by one-way ANOVA with post-Hoc Tukey's test for multiple comparisons. n = 554 cells. ***p < 0.001; **p < 0.01; ns, not significant. **d,** Representative H&E staining from C57Bl/6 mice 1, 3 or 6 weeks post-HDTVi of CAG>NICD-tdT, n = 3 mice. **e,** Schematic illustrating the experimental design to analyse entrapped hepatocytes after an extended 12-week timepoint. Created in BioRender. Hewett, C. (2026) https://BioRender.com/xjlpzpi **f,** Quantification of the percentage of tdT$^+$ cells expressing the indicated cell identity markers 12 weeks post-HDTVi of TTR>NICD-CAG>tdT in C57Bl/6 mice. TTR, Transthyretin. Data shown are means ±SD; n = 4.

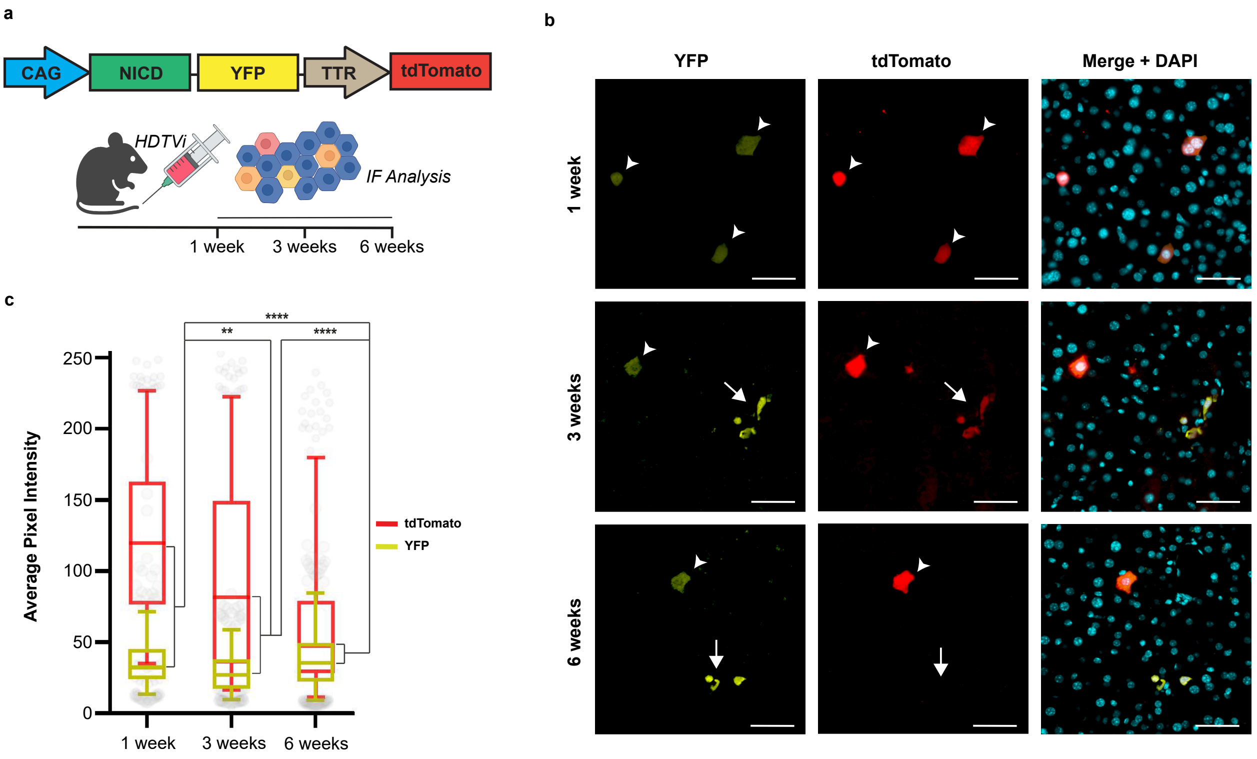

Supplementary Fig. 4. TTR promoter activity is diminished as hepatocytes progress through reprogramming to BECs.

a, Graphical depiction of the experimental procedure used to analyse the hepatocyte-specific Transthyretin (TTR) promoter activity during reprogramming. NICD and YFP expression were driven by the ubiquitous CAG promoter independent of reprogramming status while tdTomato expression was driven by the TTR promoter dependent on reprogramming status. C57Bl/6 mice were injected with the transposon by HDTVi and sacrificed at the indicated time points for liver immunofluorescence analysis. Created in BioRender. Hewett, C. (2026) https://BioRender.com/lh6a7he **b**, Representative immunofluorescence images showing YFP and tdTomato expression at the indicated time points. Scale bars, 50 μm. DAPI, 4′,6-diamidino-2-phenylindole. **c**, Quantification of the marker average pixel intensity at 1-, 3-, or 6-weeks post-HDTVi. The resulting boxplot depicts the 5th-95th percentile of values with outliers portrayed as points across two mice per timepoint. n = 1200 cells. The evolution over time of the difference between tdTomato and YFP intensities was compared using a linear mixed effects model with a likelihood-ratio test to assess statistical significance. Significance of pairwise comparisons was determined using post-hoc comparisons from the linear mixed model with Holm adjustment. ****$p < 0.0001$; **$p < 0.01$.

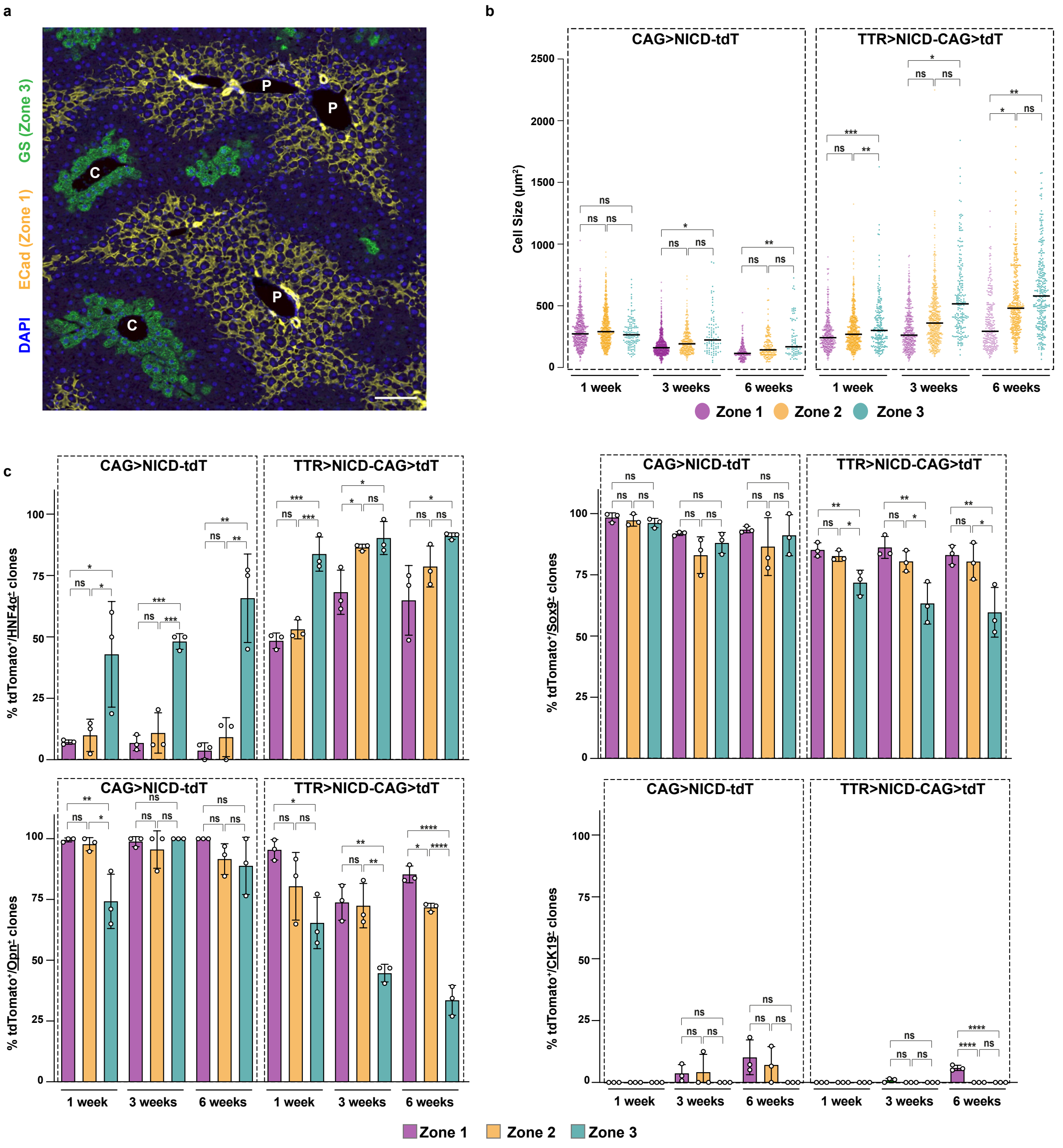

Supplementary Fig. 5. Hepatocyte reprogramming is influenced by liver zonation.
a, Example immunofluorescence image of GS and E-Cadherin staining in a normal C57Bl/6 mouse liver to indicate the method used to define zone 1 (ECadherin+/GS-), zone 2 (ECadherin-/GS-) or zone 3 (ECadherin-/GS+). Scale bar, 100μm. P, portal vein; C, central vein. **b,** Cell size analysis of tdT^{+} cells within each zone of the liver, 1-, 3- or 6-weeks post-HDTVi of the indicated transposons from the same samples in Fig.1c-e. Each data point represents a single cell. The mean of the data for each transposon/ timepoint/zone across three mice is denoted by solid black lines. n = 9631 cells. **c,**

Quantification of the same tdT^+ cells expressing the designated cell identity markers at 1-, 3- or 6-weeks post-HDTVi of the indicated transposons, within Zone 1, 2 or 3 of the liver lobule. Data displayed are means ±SD; n = 3 mice. Statistical significance was determined by one-way ANOVA followed by post-Hoc Tukey's test for multiple comparisons. ****$p < 0.0001$; ***$p < 0.001$; **$p < 0.01$; *$p < 0.05$; ns, not significant.

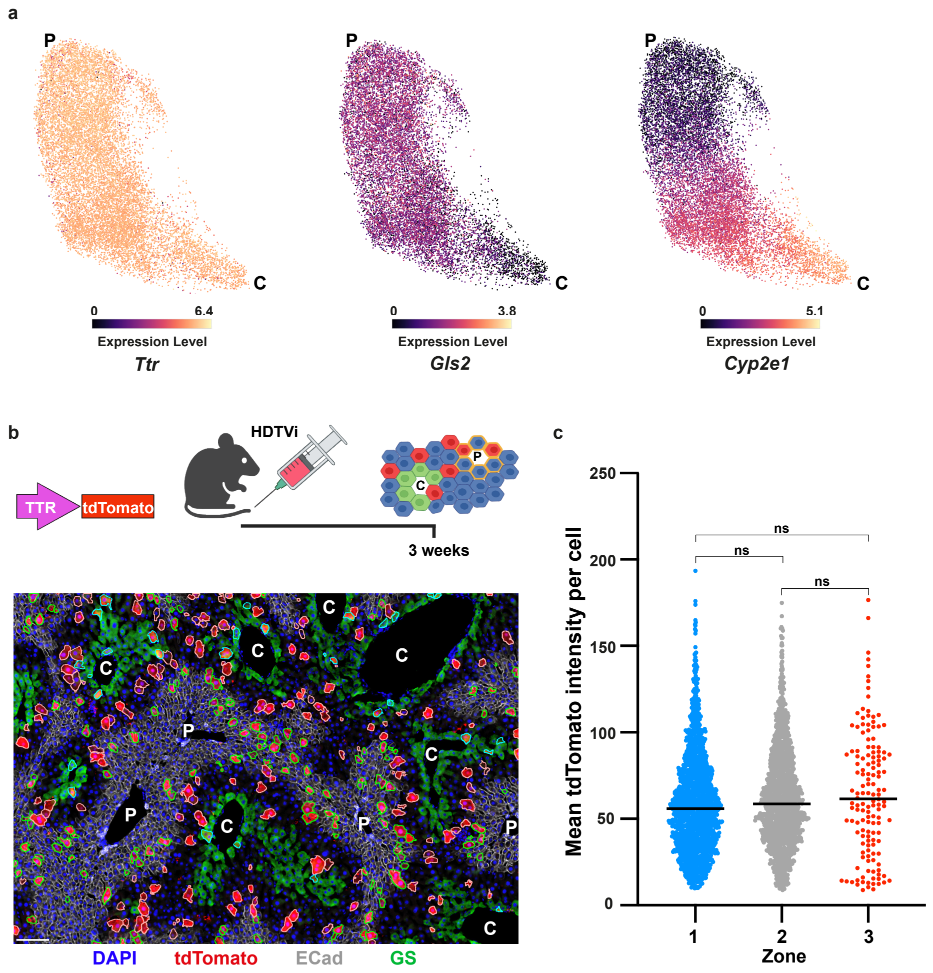

Supplementary Fig. 6. TTR activity is not biased by liver zonation.
a, UMAP plots showing gene expression levels for *Ttr*, *Gls2* (portal hepatocyte biased gene) and *Cyp2e1* (central hepatocyte biased gene) from the normal liver atlas. P indicates periportal hepatocytes, C indicates hepatocytes closest to the central vein. n = 13711 cells. **b,** Schematic illustrating the experimental design to analyse TTR driven tdTomato expression within zones 1, 2 and 3 of the liver. A representative immunofluorescence image used in this analysis is provided. Zone 1 cells are outlined in green, zone 2 cells are outlined in pink and zone 3 cells outlined in blue. Scale bar, 100mm. P, portal vein; C, central vein. Created in BioRender. Hewett, C. (2026) https://BioRender.com/ftudc6t **c,** The mean tdTomato intensity per tdT^{+} cell was analysed in three C57Bl/6 mice 3 weeks post-HDTVi of TTR>tdTomato, as described in b (solid black line denotes mean). Each data point represents a single tdT^{+} cell. n = 3174 cells. Statistical significance was determined by one-way ANOVA with post-Hoc Tukey's test for multiple comparisons. ns, not significant.

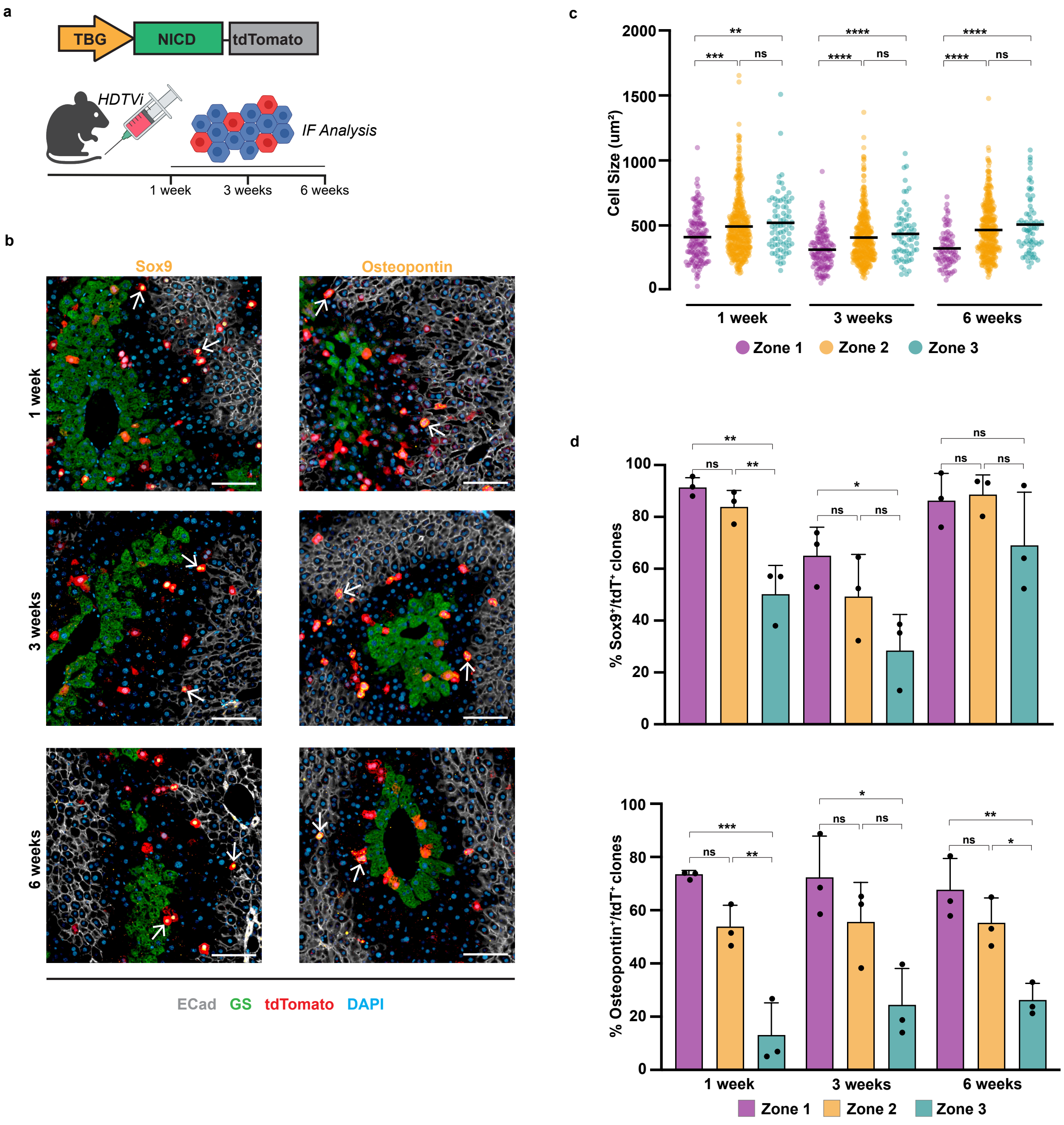

Supplementary Fig. 7. TBG driven NICD expression generates plastic states in hepatocytes with zonation bias.

a, Graphical depiction of the experimental procedure used to analyse the hepatocyte-specific Thyroxine Binding Globulin (TBG) promoter driving NICD and tdTomato. C57Bl/6 mice were injected with TBG>NICD-tdT by HDTVi and sacrificed at the indicated time points for liver immunofluorescence analysis. Created in BioRender. Hewett, C. (2026) https://BioRender.com/xjlpzpi **b**, Representative immunofluorescence images from co-staining of tdTomato with reprogramming markers, Sox9 or OPN and zonation markers ECadherin (Ecad) and Glutamine Synthetase (GS). Arrows indicate representative tdT^+ cells with reprogramming marker expression. n = 3 mice. Scale bars, 100 µm. DAPI,

4′,6-diamidino-2-phenylindole. **c**, Quantification of tdT^+ cell size at 1-, 3-, or 6-weeks post-HDTVi. Each data point represents a single cell. The mean for each time point/ zonation area across three mice is denoted by solid black or yellow lines. One-way ANOVA followed by post-Hoc Tukey's was performed to determine statistical significance. n = 1662 cells. **d**, Quantification of Sox9 and Osteopontin expression in the same tdT^+ cells. Data displayed are means ±SD; n = 3. One-way ANOVA followed by post-Hoc Tukey's was performed to determine statistical significance. ****$p < 0.0001$; ***$p < 0.001$; **$p < 0.01$; *$p < 0.05$; ns, not significant.

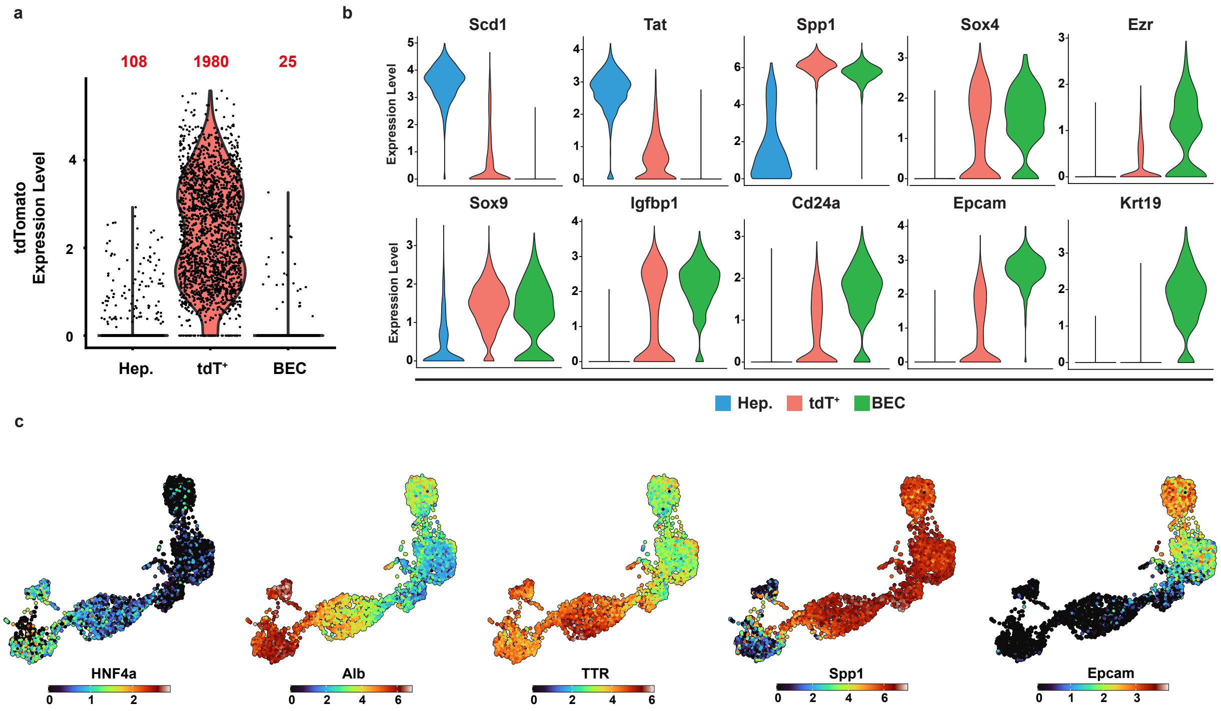

Supplementary Fig. 8. TTR>NICD-CAG>tdT generates very few terminally differentiated hepatocytes and BECs.

a, Violin plot showing the tdT expression level in each single cell within the dataset generated in Fig. 2b for the hepatocyte, tdT+ and BEC clusters. Values denote the number of tdT expressing cells within each cluster. **b,** Violin plots showing the expression level of various cell identity markers in the three clusters designated in Figure 2b. **c,** UMAP profiles for additional hepatocyte and reprogramming identity markers as in Fig. 2b. n = 3486 cells.

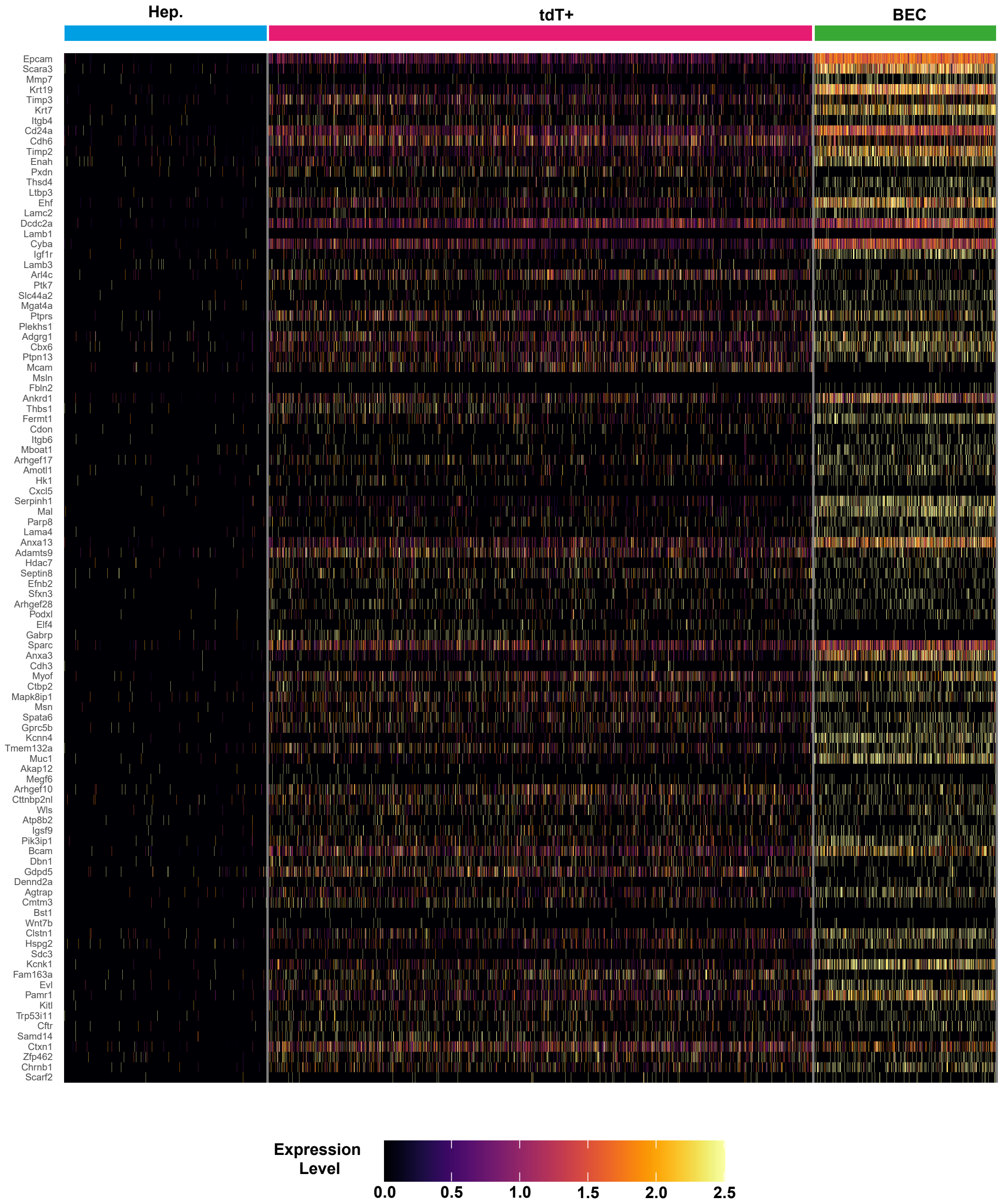

Supplementary Fig. 9. Entrapped plastic hepatocytes induce the top upregulated genes found in endogenous plastic hepatocytes.

Heatmap depicting expression levels of the top 100 genes upregulated in bulk RNA-seq of endogenous hepatocyte dataset (Merrell et al[14]), within the hepatocyte, BEC and tdT^{+} cell clusters in the dataset in Fig. 2b.

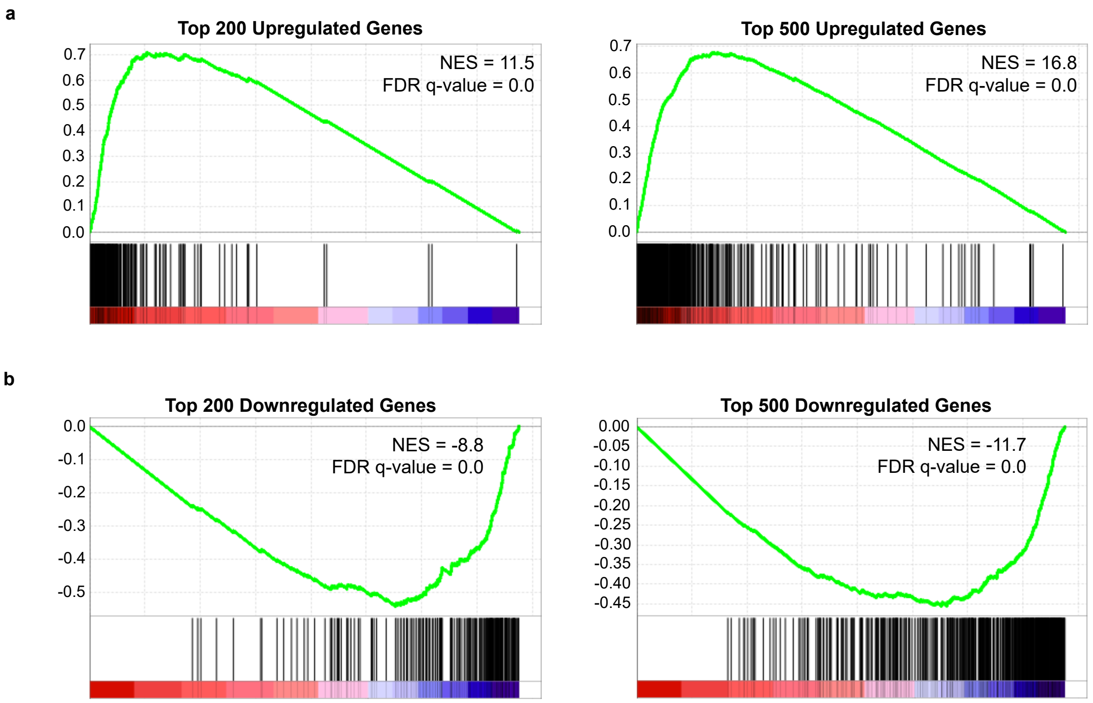

Supplementary Fig. 10. Extensive similarity of entrapped plastic hepatocytes with endogenous plastic hepatocytes.

a-b, GSEA of the top 200 and 500 genes up (a) or down-regulated (b) in Merrell et al[14] bulk RNAseq dataset, analysed on the tdT^{+} vs hepatocyte ranked gene list (x-axis).

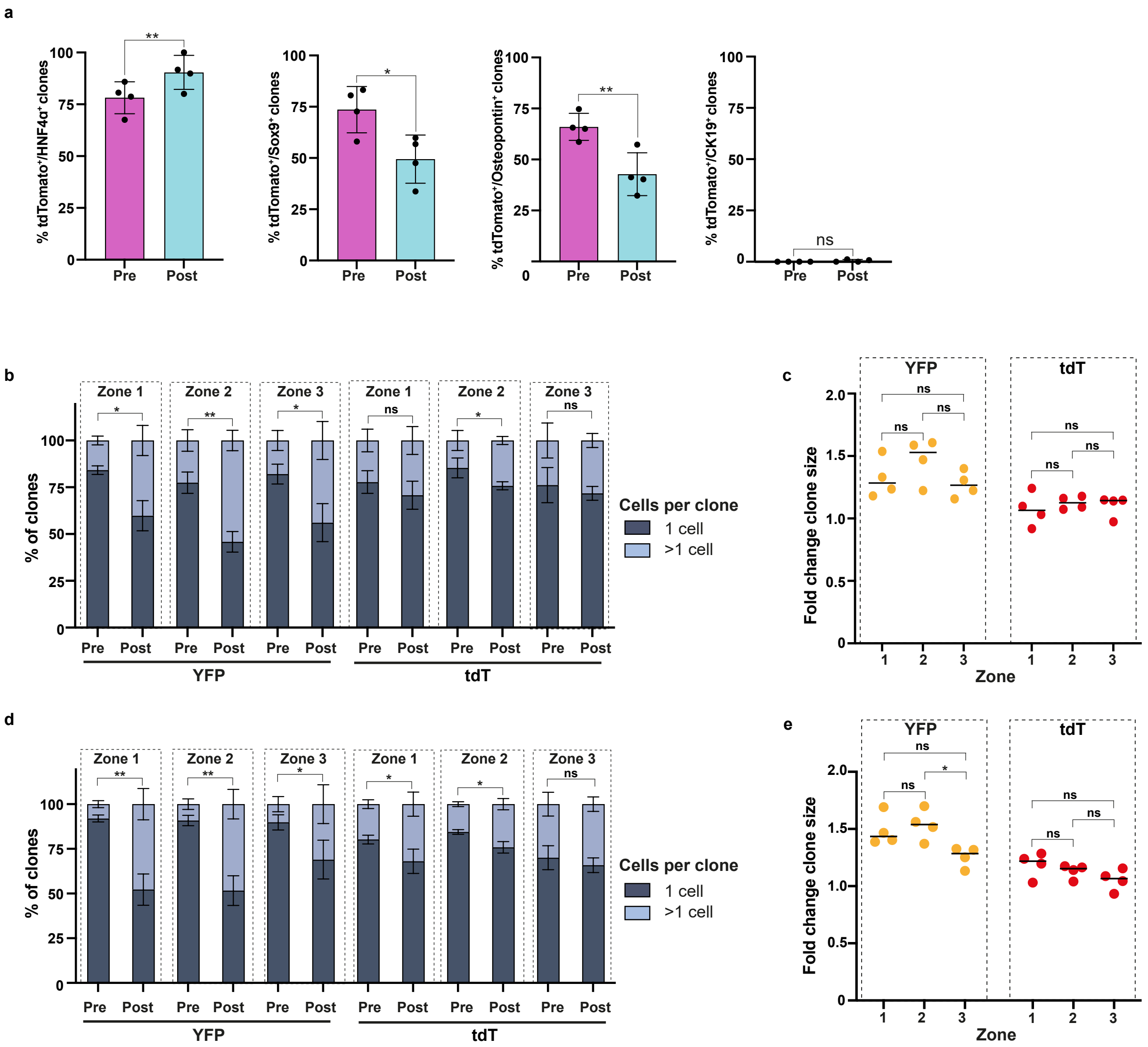

Supplementary Fig. 11. Zonation does not impact plastic hepatocyte proliferation dynamics following PHx and intermediate cell identity status is maintained.

a, Quantification of the indicated cell identity markers in tdT^+ clones on the pre- and post-PHx samples from the experiment described in Figure 3a. Statistical significance was determined by paired, two-tailed t-test. **b,** Quantification of the number of cells per YFP^+ and tdT^+ clone found within each zone of the liver was performed on pre- and post-PHx samples from the experiment described in Figure 3a. Data shown is mean ±SD. n = 4 mice. **c,** The fold change in clone size from the same clones quantified in (a) is shown. Lines denote the mean of the data. Paired, two-tailed t-test was performed to determine statistical significance. **d,e** Zonation analysis of the experiment shown in Figure 3e as described in (a, b). Statistical significance was determined by paired, two-tailed t-test (b, d) or one-way ANOVA with Tukey's correction for multiple comparisons (c,e). $^{**}p < 0.01$; $^{*}p < 0.05$; ns, not significant.

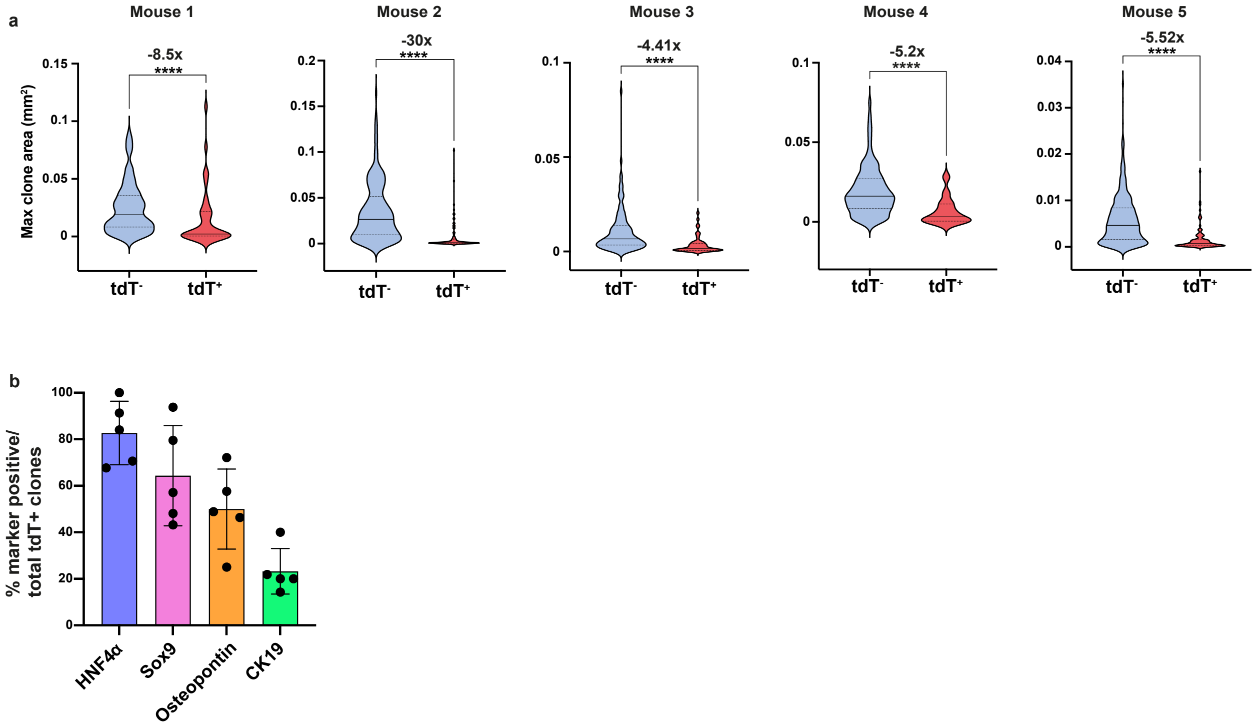

Supplementary Fig. 12. Plastic hepatocyte proliferation and reprogramming status in the Fah$^{-/-}$ model of liver repopulation.

a, Violin plots from 5 individual Fah$^{-/-}$ recipients transplanted with a combination of tdT^{-} (normal) hepatocytes and tdT^{+} (plastic hepatocytes) showing the maximum clone area of every individual tdT^{-} or tdT^{+} clone within 10 serial sections. Dashed lines represent the 25th and 75th percentiles of the data; solid line represents the median. Statistical significance was determined by unpaired, two-tailed t-test. n = 1351 clones. ****p<0.0001 **b,** Quantification of the percentage of tdT^{+} clones expressing the indicated cell identity markers in the same mice from (a). n = 5 mice.

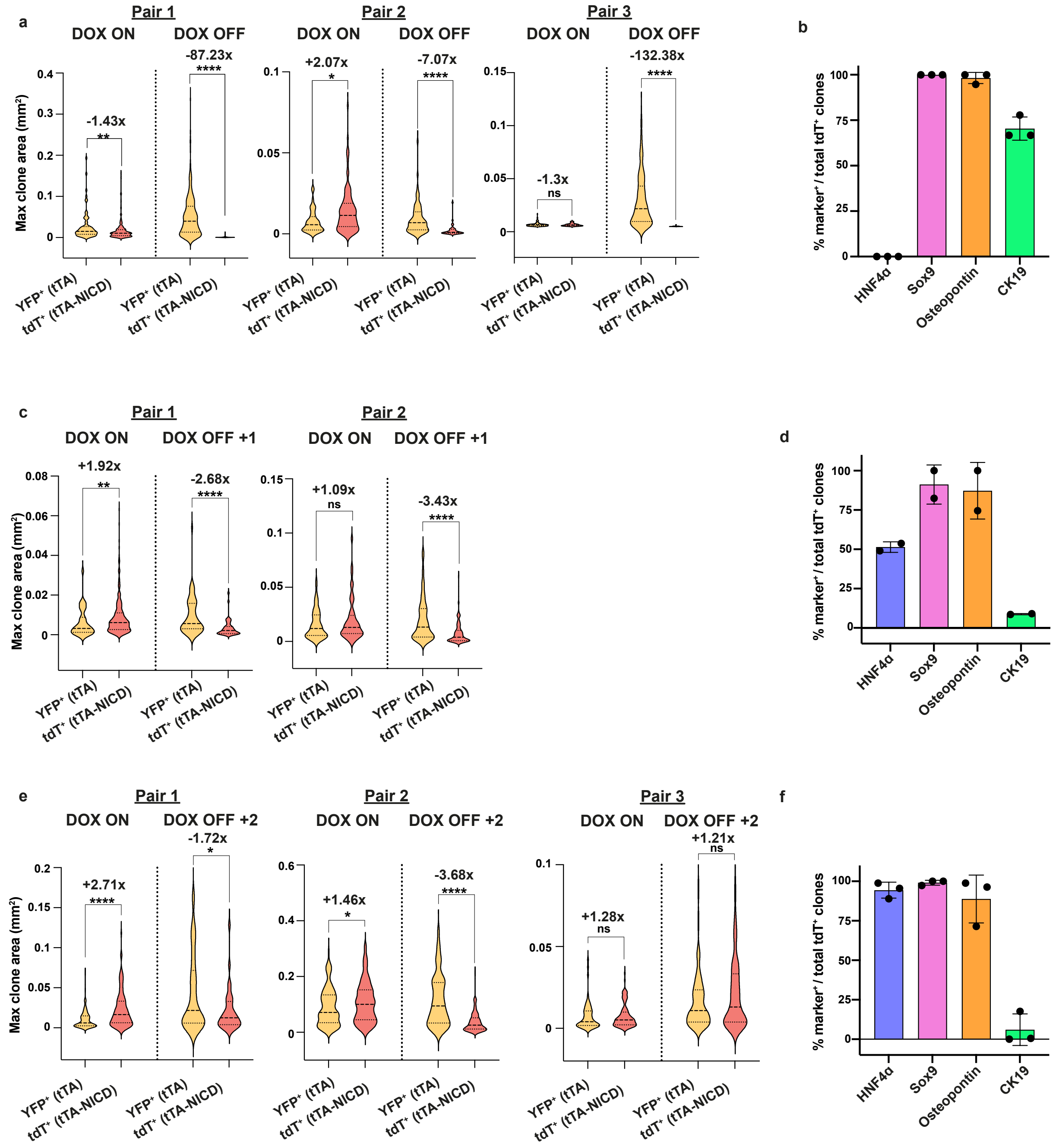

Supplementary Figure 13. Manipulation of reprogramming levels after transplantation and resulting repopulation capacity in FRG mice.

a, Violin plots from 3 individual pairs of FRG recipients transplanted with a combination of tdT⁺ (LAP-tTA; Tet-Off NICD) and YFP⁺ (LAP-tTA) donor hepatocytes and maintained with or without doxycycline (Dox), showing the mean max clone area as measured across 10 serial sections. Fold change in median area between tdT⁺ and YFP⁺ clones in each mouse is provided. n = 1311 clones. **b,** Quantification of the percentage of tdT⁺ clones expressing the indicated cell identity markers in the same mice from (a). Every

clone was analysed across 5 serial sections. n = 3 pairs. **c,d** Analysis of FRG recipient mice, readministered Dox halfway through the repopulation period, as described in (a, b). n = 780 clones, 2 pairs. **e,f** Analysis as in (a,b), of FRG recipient mice readministered Dox twice during the repopulation phase (every 20 days). n = 1227 clones, 3 pairs. Statistical significance in a,c,e was determined by unpaired, two-tailed t-test. ****p<0.0001; ***p<0.001; **p<0.001; *p<0.05; ns, not significant.

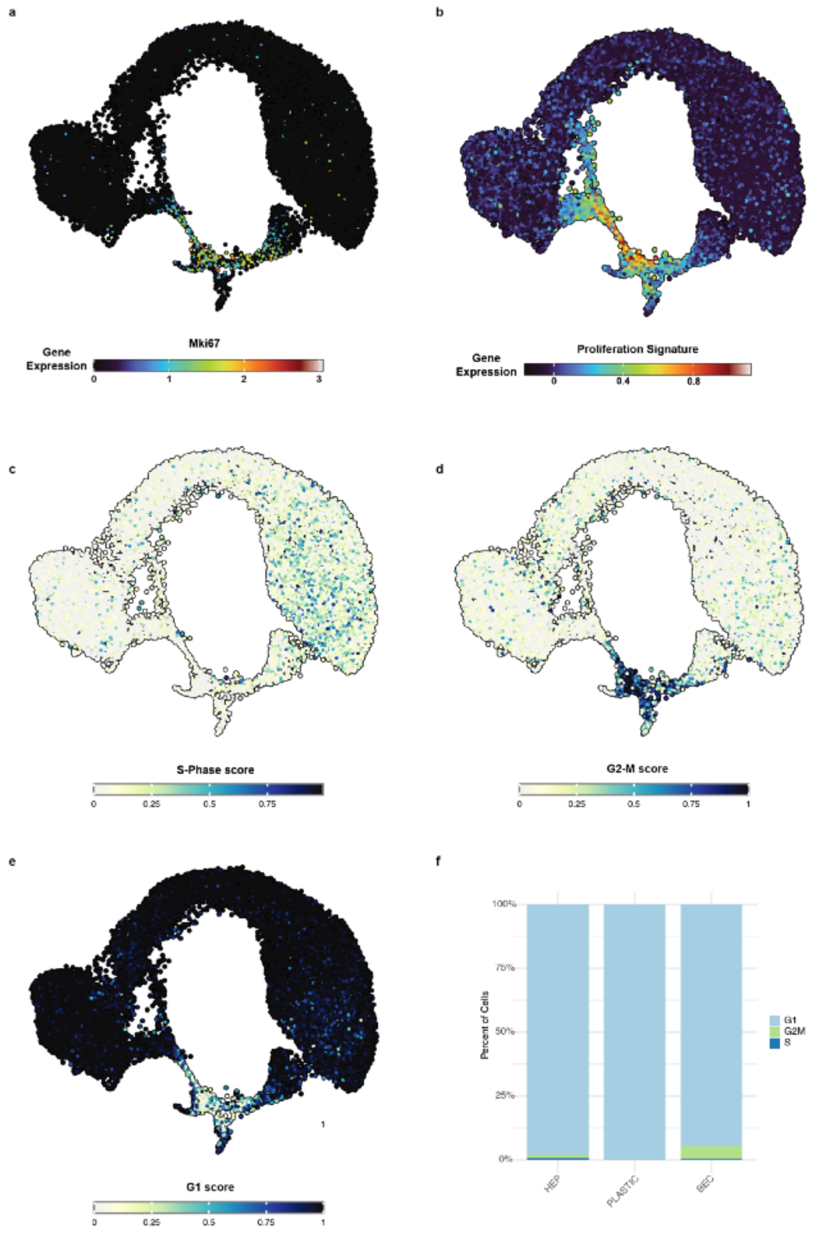

Supplementary Fig. 14. Proliferation is restricted to terminally differentiated hepatocytes and BECs during liver damage and regeneration.

a, UMAP plot showing expression of the proliferation marker Ki67 in the integrated dataset as in Fig.2c. **b,** UMAP plot showing enrichment of a proliferative gene signature (*Ki67, Pcna, Cdk1, Mcm2, Mcm3, Mcm4, Mcm5, Mcm6, Mcm7, Ccnb1, Aurkb, Cenpf, Top2a*) in the same dataset. **c-e**, UMAP plots displaying the continuous Cyclone G1-, S- and G2/M-phase scores. n = 21420 cells. **f**, Stacked bar chart of Cyclone-inferred cell-cycle phases in each of the three cell clusters defined in Figure 2c.

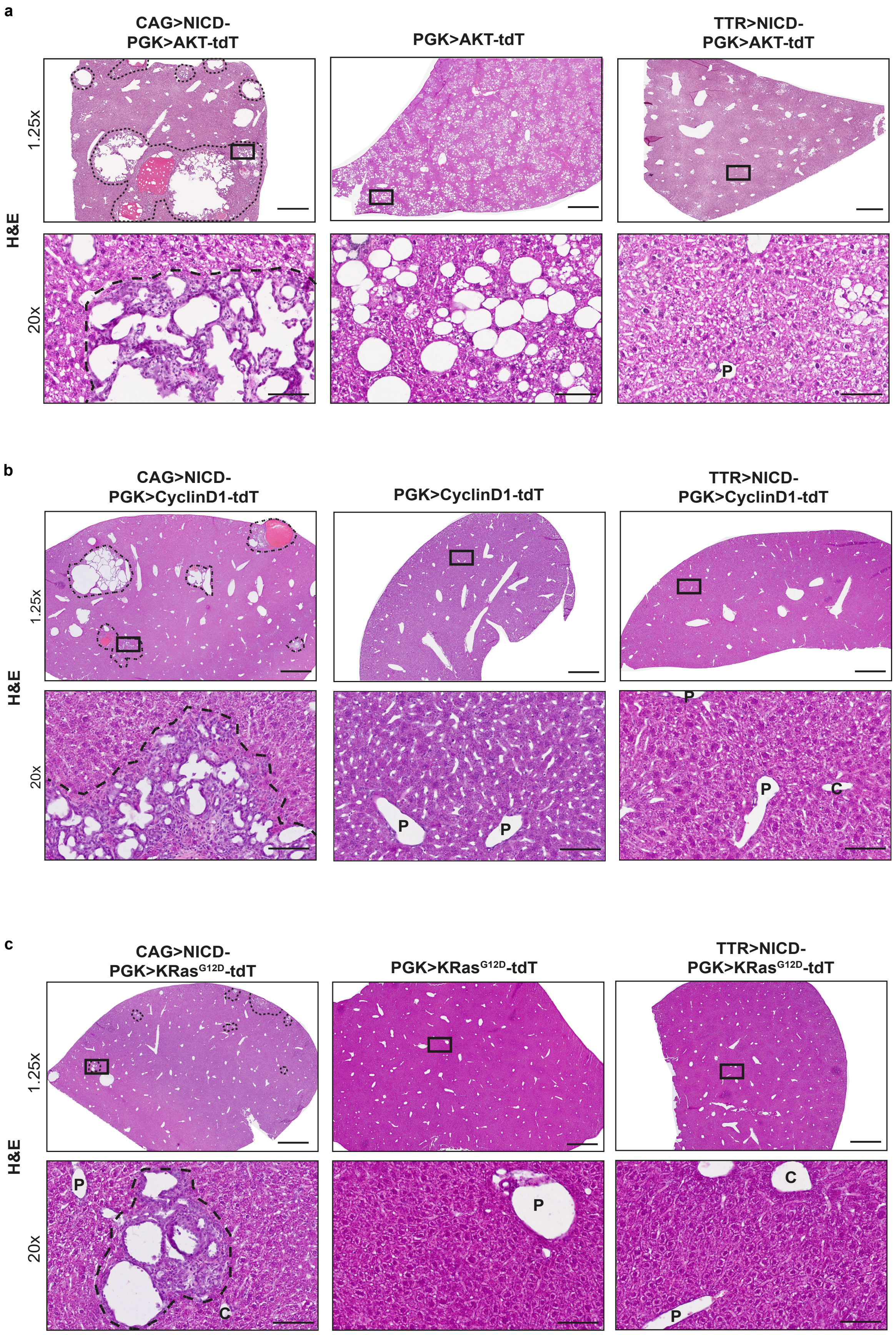

a
CAG>NICD-
PGK>AKT-tdT
PGK>AKT-tdT
TTR>NICD-
PGK>AKT-tdT
1.25x
H&E
20x
P
b
CAG>NICD-
PGK>CyclinD1-tdT
PGK>CyclinD1-tdT
TTR>NICD-
PGK>CyclinD1-tdT
1.25x
H&E
20x
P
P
P
P
C
c
CAG>NICD-
PGK>KRasG12D-tdT
PGK>KRasG12D-tdT
TTR>NICD-
PGK>KRasG12D-tdT
1.25x
H&E
20x
P
C
P
C
P

Supplementary Fig. 15. Plastic hepatocyte states limit tumorigenesis in models of liver cancer development.

a-c, Representative images from H&E staining of livers from C57Bl/6 mice HDTV-injected with the indicated transposons, as described in Fig. 5. Visible cholangiocarcinoma tumours (observed in CAG>NICD-PGK>oncogene-tdT) are demarcated by dashed black lines. Solid black boxes indicate area shown at higher magnification. Scale bars 1.25x magnification, 1mm; Scale bars 20x magnification, 100mm.

a

TTR>NICD-PGK>AKT*-tdT

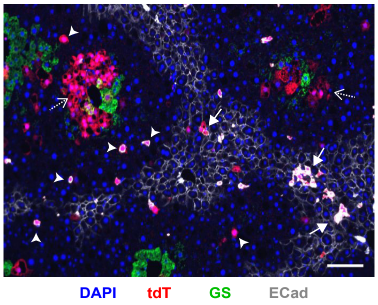

b

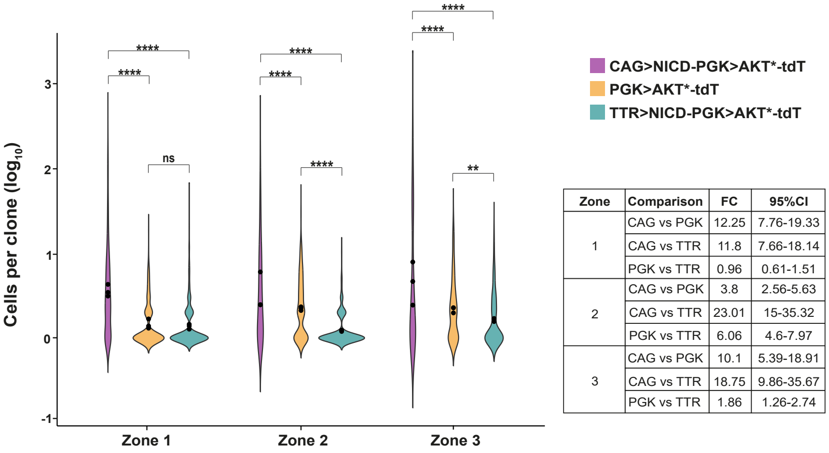

Zone	Comparison	FC	95%CI
1	CAG vs PGK	12.25	7.76-19.33
	CAG vs TTR	11.8	7.66-18.14
	PGK vs TTR	0.96	0.61-1.51
2	CAG vs PGK	3.8	2.56-5.63
	CAG vs TTR	23.01	15-35.32
	PGK vs TTR	6.06	4.6-7.97
3	CAG vs PGK	10.1	5.39-18.91
	CAG vs TTR	18.75	9.86-35.67
	PGK vs TTR	1.86	1.26-2.74

c

TTR>NICD-PGK>CyclinD1-tdT

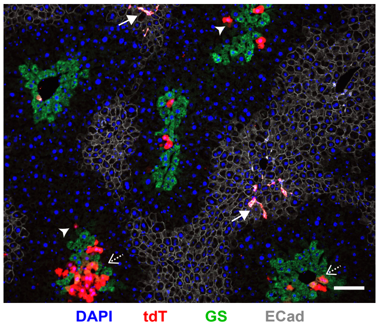

d

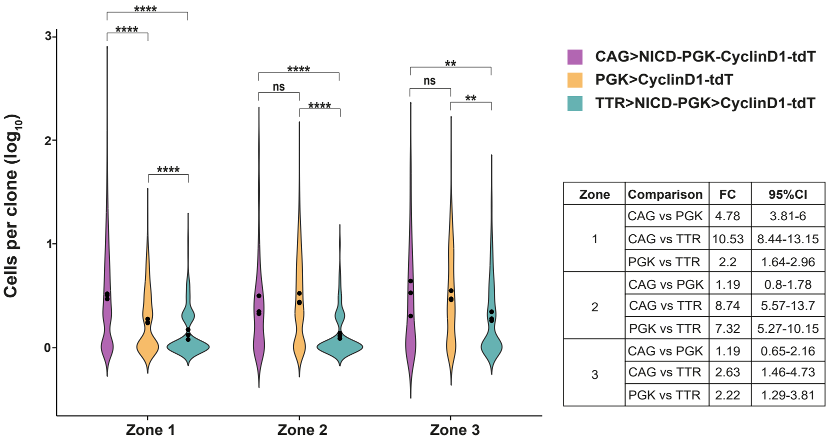

Zone	Comparison	FC	95%CI
1	CAG vs PGK	4.78	3.81-6
	CAG vs TTR	10.53	8.44-13.15
	PGK vs TTR	2.2	1.64-2.96
2	CAG vs PGK	1.19	0.8-1.78
	CAG vs TTR	8.74	5.57-13.7
	PGK vs TTR	7.32	5.27-10.15
3	CAG vs PGK	1.19	0.65-2.16
	CAG vs TTR	2.63	1.46-4.73
	PGK vs TTR	2.22	1.29-3.81

e

TTR>NICD-PGK>$KRas^{G12D}$-tdT

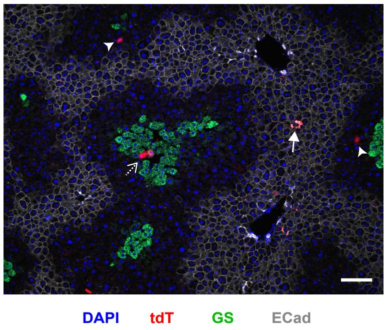

f

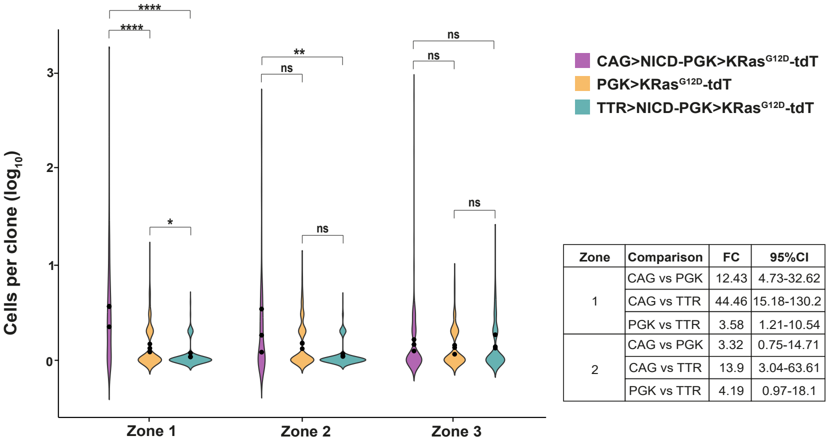

Zone	Comparison	FC	95%CI
1	CAG vs PGK	12.43	4.73-32.62
	CAG vs TTR	44.46	15.18-130.2
	PGK vs TTR	3.58	1.21-10.54
2	CAG vs PGK	3.32	0.75-14.71
	CAG vs TTR	13.9	3.04-63.61
	PGK vs TTR	4.19	0.97-18.1

Supplementary Fig. 16. Suppression of oncogene-induced proliferation by plastic states in hepatocytes is influenced by zonation.

a, Example of immunofluorescence staining of tdT, ECad (Zone 1) and GS (Zone 3) on liver sections from C57Bl/6 mice 12 weeks post-HDTVi of TTR>NICD-PGK>AKT*-tdT. Arrowheads denote tdT^+ single cell clones; solid arrows indicate tdT^+ proliferating clones in zone 1; dashed arrows indicate tdT^+ proliferating clones in zone 3. Scale bars, 100mm. ECad = Ecadherin; GS = glutamine synthetase. **b,** Violin plot showing the tdT^+ clone size (cells per clone) generated by the indicated transposons within each zone of the liver, 8 weeks post-HDTVi. Dots represent the mean value from individual mice. Within each zone, statistical analysis was performed by fitting a negative binomial model that featured random effects for each mouse and an additive fixed effect for the three groups (CAG/PGK/TTR). Statistical significance of overall differences between the three groups was assessed with a likelihood-ratio test. Subsequently, pairwise comparisons between groups were conducted using estimated marginal means with Tukey adjustment for multiple comparisons. Fold changes (FC) and 95% confidence intervals (CI) are shown for the designated comparisons. n=3945 clones. **c-f** Analysis of C57BL/6 mice 13 weeks post-HDTVi of TTR>NICD-PGK>CyclinD1-tdT (c, d) or 11 weeks post-HDTVi of TTR>NICD-PGK>$KRas^{G12D}$-tdT (e,f) as described in (a,b). n = (d) 4831 clones, (f) 4211 clones. ****$p < 0.0001$; **$p<0.01$; ns, not significant.

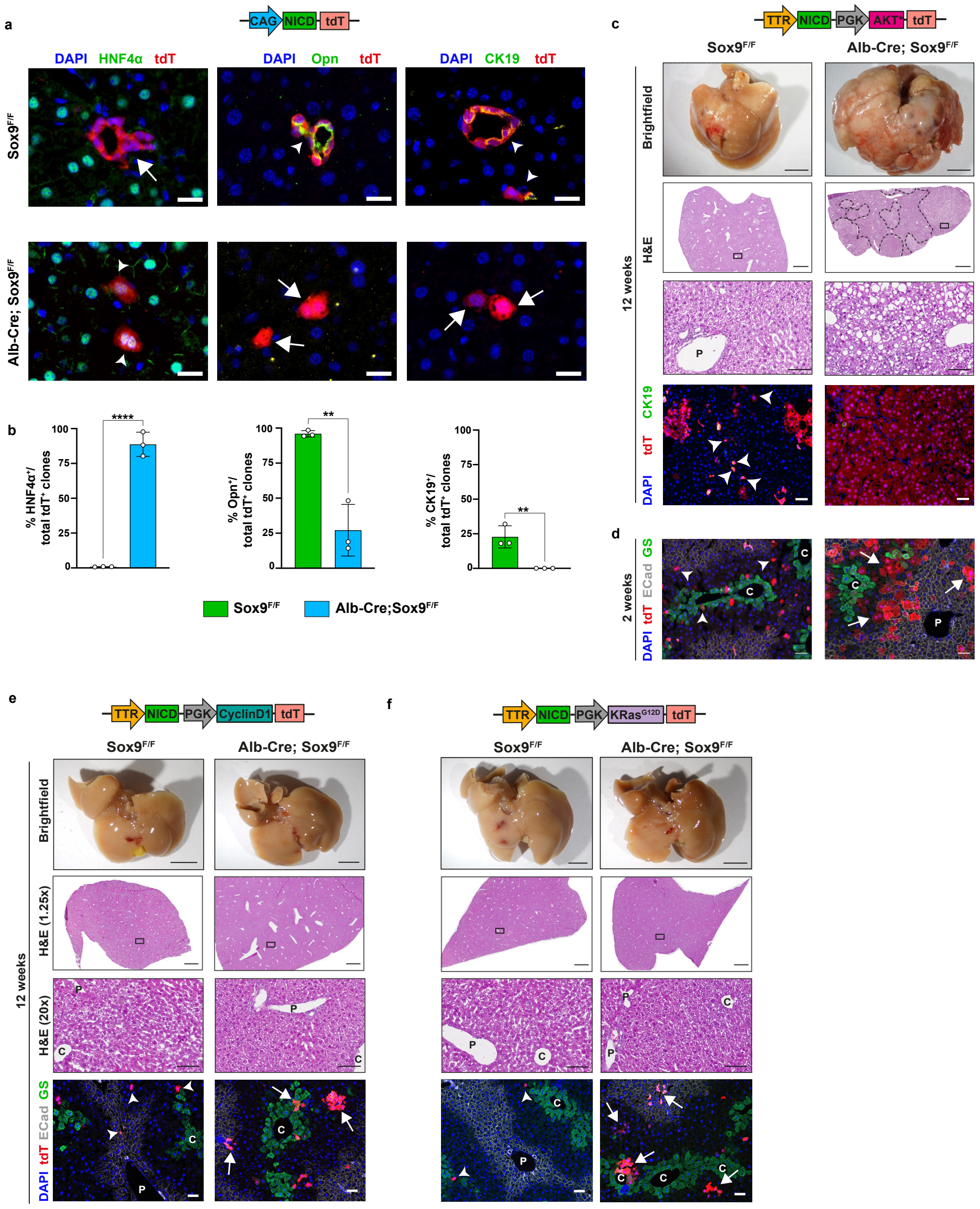

Supplementary Fig. 17. Inhibition of hepatocyte reprogramming licenses oncogene-induced proliferation.

a, Example images from immunofluorescence of liver sections from Alb-Cre; Sox9$^{F/F}$ and Sox9$^{F/F}$ mice 6 weeks after HDTVi of CAG>NICD-tdT. Liver sections were stained for tdT in combination with either HNF4a, Opn or CK19. n = 3 mice. **b,** Quantification of the percentage of tdT^{+} clones expressing the indicated cell identity marker, corresponding to samples in (a). Data displayed are means ±SD; n = 3. Statistical significance was determined by unpaired, two-tailed t-test. ****p<0.0001; **p<0.01. **c,** Whole liver photos, H&E and representative images of immunofluorescence of liver sections for tdT and CK19, from Sox9$^{F/F}$ or Albumin-Cre; Sox9$^{F/F}$ mice 12 weeks after

HDTVi of TTR>NICD-PGK>AKT*tdT. Visible tumors are demarcated by dotted black lines. Solid black boxes indicate area shown at higher magnification. Arrowheads denote tdT^+ single cell clones. Scale bars whole livers, 0.5cm; Scale bars, H&E 1.25x, 1mm; Scale bars, H&E 20x, 100µm. Scale bars immunofluorescence, 50mm. n = 3 mice. **d,** Representative immunofluorescence staining of tdT, E-Cad and GS 2 weeks post-HDTVi of TTR>NICD-PGK>AKT*-tdT in $Sox9^{F/F}$ or Alb-Cre; $Sox9^{F/F}$ mice. Arrowheads denote examples of tdT^+ single cell clones within each zone of the liver; arrows show examples of tdT^+ proliferating clones within all 3 zones. P, portal vein; C, central vein. Scale bars, 50mm. n = 3 mice. **e,f,** Analysis of $Sox9^{F/F}$ or Alb-Cre; $Sox9^{F/F}$ mice, 12 weeks post-HDTVi of TTR>NICD-PGK>CyclinD1-tdT (e) TTR>NICD-PGK>$KRas^{G12D}$-tdT (f) as described in (c-d). n = 3 mice.

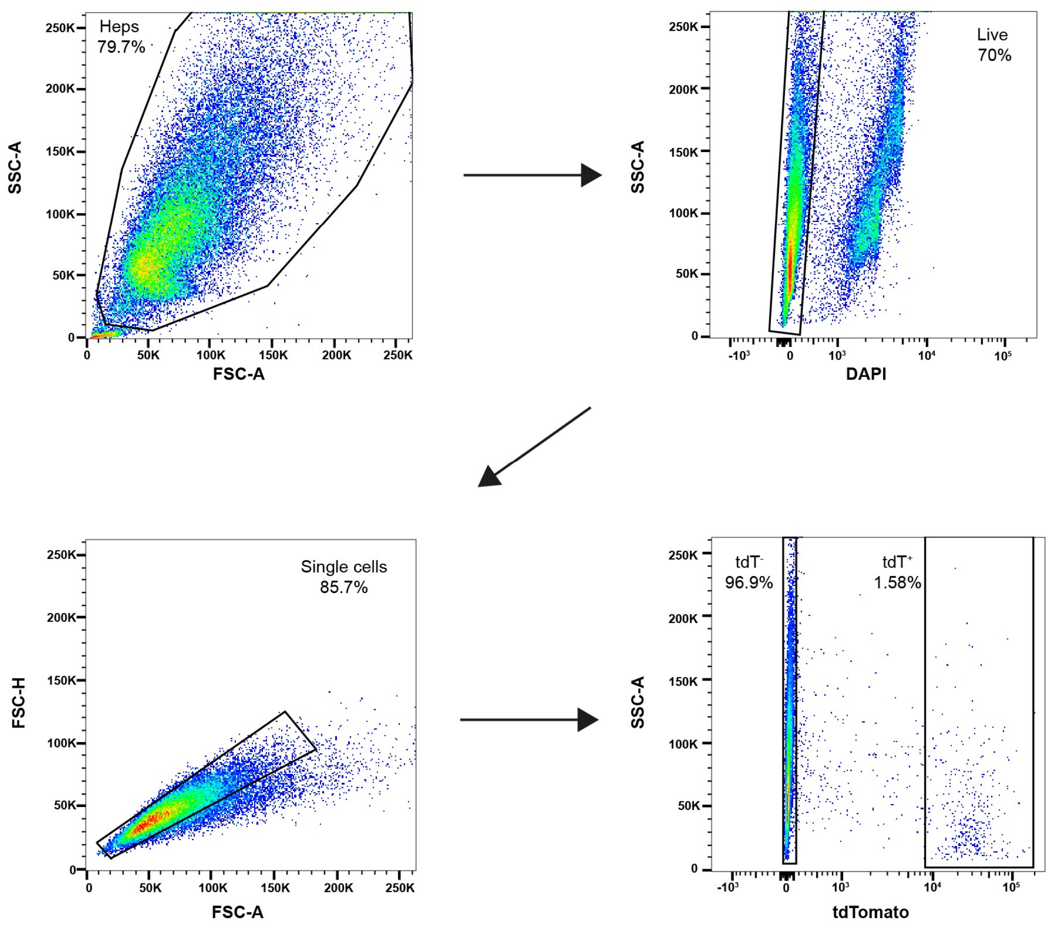

Supplementary Fig. 18. Flow cytometry gating strategy for analysis and sorting of tdT$^-$ and tdT$^+$ mouse hepatocyte populations.

Example of the gating strategy used for analysis and sorting of hepatocyte populations from the hepatocyte fraction isolated from the mouse liver.

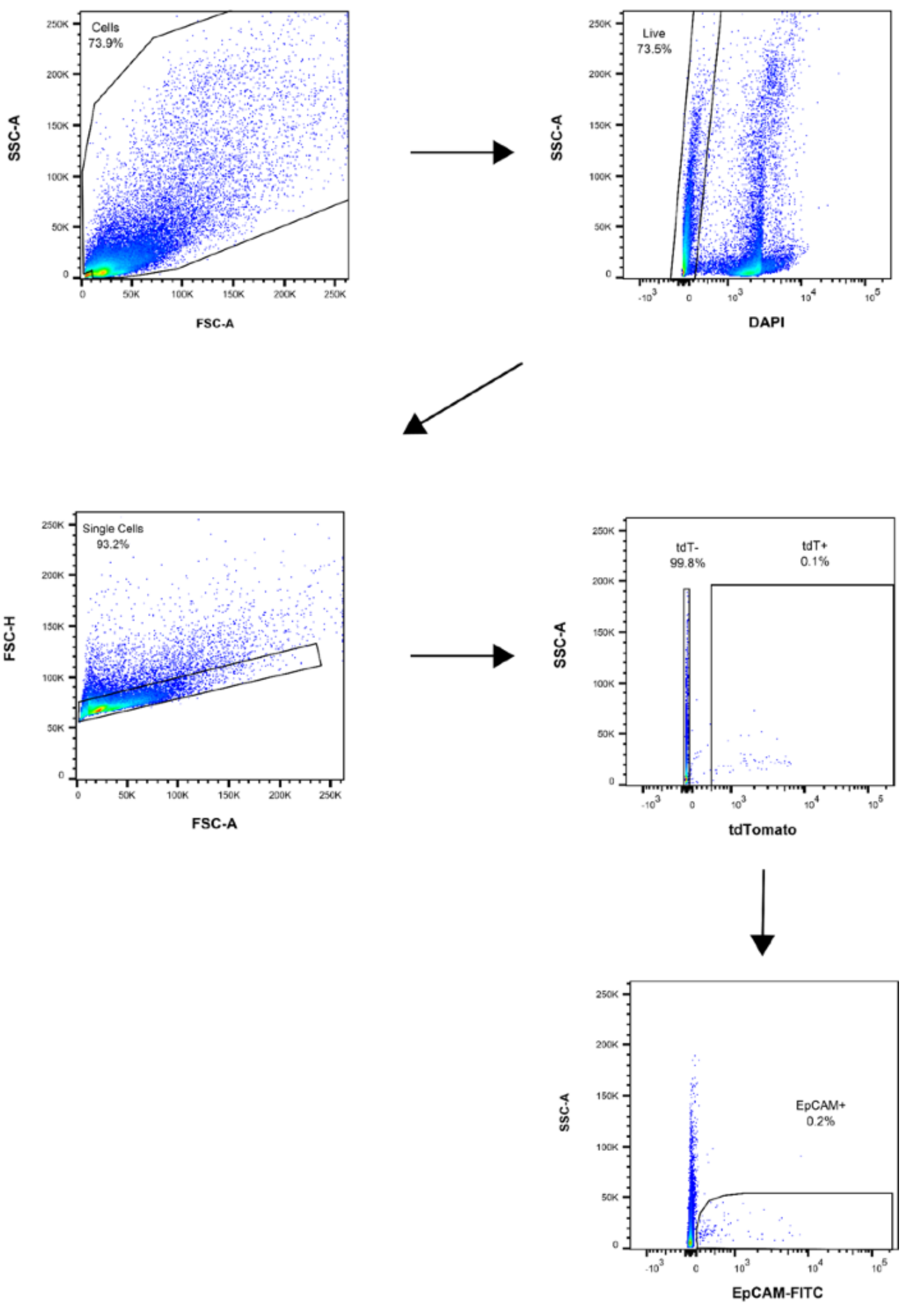

Supplementary Fig. 19. Flow cytometry gating strategy for sorting mouse BEC population.

Example of the gating strategy used for sorting of tdT$^-$ cells and tdT$^-$/EpCAM$^+$ BECs from the supernatant fraction obtained during mouse liver cell isolation.

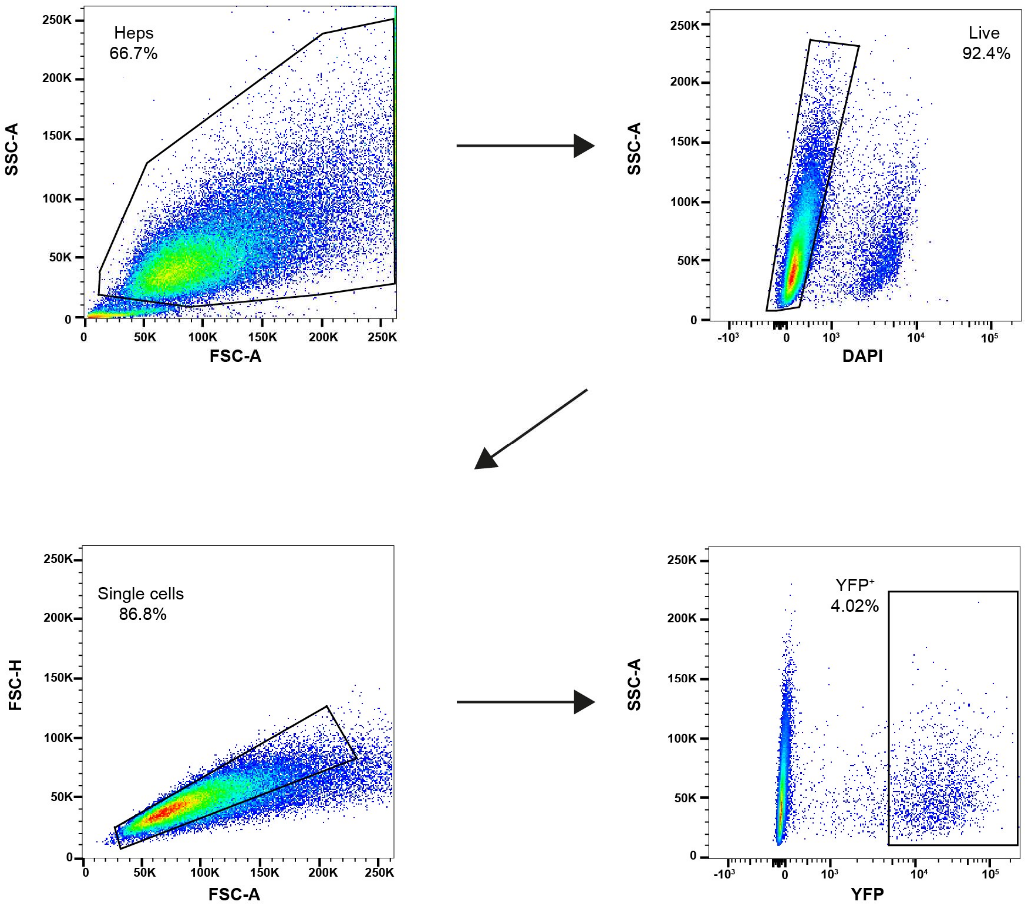

Supplementary Fig. 20. Flow cytometry gating strategy for analysis and sorting of YFP⁺ mouse hepatocyte populations.

Example of the gating strategy used for analysis and sorting of YFP⁺ hepatocytes from the hepatocyte fraction of the mouse liver.
